# Mac-2 binding protein glycosylation isomer is a potential biomarker to predict portal hypertension and bacterial infection in cirrhotic patients

**DOI:** 10.1371/journal.pone.0258589

**Published:** 2021-10-14

**Authors:** Pei-Shan Wu, Yun-Cheng Hsieh, Kuei-Chuan Lee, Yi-Hsiang Huang, Ming-Chih Hou, Han-Chieh Lin

**Affiliations:** 1 Division of Gastroenterology and Hepatology, Department of Medicine, Taipei Veterans General Hospital, Taipei, Taiwan; 2 Department of Medicine, School of Medicine, National Yang Ming Chiao Tung University, Taipei, Taiwan; 3 Endoscopy Center for Diagnosis and Treatment, Taipei Veterans General Hospital, Taipei, Taiwan; National Taiwan University Hospital, TAIWAN

## Abstract

**Objectives:**

Mac-2-binding protein glycosylation isomer (M2BPGi) is a novel plasma biomarker for liver fibrosis, but less is known about its role in portal hypertension. We aimed to evaluate the association between M2BPGi and hepatic venous pressure gradient (HVPG) and to investigate its predictive value on prognosis of cirrhotic patients.

**Methods:**

Forty-eight cirrhotic patients who underwent HVPG measurement in Taipei Veterans General hospital were retrospectively enrolled. The Spearman’s correlation test was used to analyze the correlation between plasma M2BPGi levels and HVPG and other parameters. Cox proportional hazards regression models were used to identify predictors for clinical outcomes.

**Results:**

Plasma M2BPGi levels were higher in cirrhotic patients than healthy subjects and significantly correlated with HVPG levels (r_s_ = 0.45, *p* = 0.001). On multivariate Cox regression analysis, higher plasma M2BPGi levels [≥ 6 cut-off index (C.O.I)] did not predict mortality within five years for cirrhotic patients and the result was similar in patients without hepatocellular carcinoma. Interestingly, M2BPGi ≥ 6 C.O.I was a potential predictor of bacterial infection within five years [Hazar ratio (HR) = 4.51, *p* = 0.003]. However, M2BPGi failed to predict occurrence of other cirrhosis-related complications, including variceal bleeding, ascites formation, spontaneous bacterial peritonitis, hepatorenal syndrome and hepatic encephalopathy.

**Conclusion:**

Plasma M2BPGi levels positively correlated with HVPG and higher serum M2BPGi levels might have a potential role in predicting development of bacterial infection for cirrhotic patients with portal hypertension.

## Introduction

Portal hypertension is a major and unfavorable consequence of liver cirrhosis. Portal pressure is determined indirectly by measurement of the hepatic venous pressure gradient (HVPG), the difference between the wedged hepatic venous pressure and the free hepatic venous pressure, via hepatic vein catheterization [1]. With the progression of hepatic fibrosis, portal pressure increases and then progresses to clinically significant portal hypertension (HVPG at or above 10mmHg), which is an important cut-off value for developing cirrhosis-related complications, such as ascites formation, variceal bleeding, hepatic encephalopathy, and hepatic decompensation [2,3]. Therefore, evaluation of the presence and degree of portal hypertension in cirrhotic patients is a critical issue to avoid adverse clinical outcomes.

Currently, HVPG measurement has been used clinically for predicting events of clinical decompensation in patients with compensatory cirrhosis, monitoring response of non-selective beta-blockers, and assessing the risk of liver failure after liver resection in patients with chronic liver disease or cirrhosis [4–6]. Though HVPG measurement is considered a safe procedure, the invasiveness of this procedure limits its clinical applications for cirrhotic patients. Consequently, non-invasive diagnostic tools have emerged as alternatives to assess liver fibrosis and portal hypertension.

Recently, Mac-2-binding protein glycosylation isomer (M2BPGi) has been introduced as a novel biomarker for liver fibrosis [7,8]. Emerging studies have demonstrated that M2BPGi is a potential predictor for hepatic fibrosis of various etiologies, such as viral hepatitis, nonalcoholic fatty liver disease, autoimmune hepatitis, and primary biliary cirrhosis and biliary atresia [9]. Its plasma concentration reflects the effect of antiviral treatment in both chronic hepatitis B and hepatitis C and plays a role in risk evaluation of hepatocellular carcinoma development in viral hepatitis [9,10]. M2BPGi is also a surrogate marker for postoperative ascites after receiving resection of hepatocellular carcinoma [11]. Higher plasma levels of M2BPGi were also associated with poor prognosis in cirrhotic patients [12]. Although M2BPGi levels have good correlation with the degree of liver fibrosis, there is limited study to investigate the predictive role of M2BPGi in complications and prognosis in cirrhotic patients with portal hypertension.

In this study, we aimed to investigate the association between M2BPGi and HVPG and the role of M2BPGi in predicting cirrhosis-related complications in portal hypertension.

## Materials and methods

### Patients

From April 2000 to July 2020, forty-eight cirrhotic patients who underwent hemodynamic studies for evaluation of the presence and degree of portal hypertension at Taipei Veterans General hospital were retrospective enrolled. Written informed consent was obtained from each patient before hemodynamic study. This study has been approved by the Institutional Review Board of Taipei Veterans General Hospital (IRB No., 2021-02-012CC) and follows the tenants of the Declaration of Helsinki.

The diagnosis of liver cirrhosis was based on clinical, biochemical, and imaging findings for all patients. Patients were excluded if they have portal vein thrombosis, hepatocellular carcinoma or other liver tumor, active infection, active variceal bleeding, active alcohol drinking, hepatic encephalopathy, use of non-selective beta-blockers or vasoactive drugs, history of previous transjugular intrahepatic portosystemic shunt placement or previous operations for portal hypertension.

Demographic characteristics, laboratory data, and medical history were collected retrospectively by reviewing patients’ medical records. Severity of underlying liver disease was measured using the Child-Pugh score and Model for End-Stage Liver Disease (MELD) score. Patients were followed until death, liver transplant or until five years of follow-up. During the follow-up period, clinical events were recorded, which included (1) complication of cirrhosis, such as spontaneous bacterial peritonitis (SBP), hepatic encephalopathy, newly developed ascites, hepatorenal syndrome, first or recurrent variceal bleeding, (2) newly diagnosed hepatocellular carcinoma, (3) events of bacterial infection, including SBP, (4) death, and (5) liver transplant.

### Hemodynamic measurements

The enrolled patients underwent hemodynamic evaluation after overnight fasting. Under local anesthesia, a 7-Fr Swan–Ganz thermodilution catheter (Viggo-Spectramed, Oxnard, CA, USA) was placed in the right jugular vein or right femoral vein and was then advanced into the hepatic vein using the Seldinger technique as previous described [13]. The free hepatic venous pressure and wedge hepatic venous pressure were measured by inflation and deflation of the balloon with a multichannel recorder (model 78534C, Hewlett Packard, Palo Alto, CA). HVPG was calculated as the difference between wedged hepatic venous pressure and free hepatic venous pressure. After hepatic vein catheterization, the catheter was then advanced into right side of heart and the pulmonary artery to measure systemic hemodynamics, including right arterial pressure (RAP), mean pulmonary arterial pressure (MPAP), and pulmonary capillary wedge pressure (PCWP). Mean arterial pressure (MAP). Cardiac output (CO) was measured by using the thermodilution method. Heart rate and mean artery pressure (MAP) were recorded with an external vital sign monitor (Dinamap 8100, Critikon, Tampa, FL). Systemic vascular resistance (SVR; dynes•s/cm5) was calculated using the following equation: 80 x (MAP—RAP)/CO.

### Biochemical analysis

The blood samples were obtained from patients during hemodynamic evaluation. Plasma M2BPGi levels were measured by sandwich immunoassay with the HISCL M2BPGi regent (Sysmex Co., Kobe, Japan) using a fully automated immunoassay machine, HISCL-800 (Sysmex Co., Kobe, Japan). The measured M2BPGi values were indexed with the obtained values using the following equation: Cut-off index (C.O.I) = ([M2BPGi]_sample_−[M2BPGi]_negative control_)/([M2BPGi]_positive control_−[M2BPGi]_negative control_), where [M2BPGi]_sample_ represents patients’ plasma levels of M2BPGi. A calibrator solution that was preliminarily standardized to yield a C.O.I of 1.0 was used as a positive control and the buffer was used as a negative control [14]. Plasma interleukin-1α (IL-1α), interleukin-1β (IL-1β), interleukin-1 receptor antagonist (IL-1Ra) levels were measured by enzyme-linked immunosorbent assay (BioLegend [San Diego, CA,USA] for IL-1 α and IL-1 β, and eBioscience [San Diego,CA, USA] for IL-1Ra) as previously described [13]. Plasma levels of endotoxin and nitric oxide were also measured by enzyme-linked immunosorbent assay (Cayman Chemical, Ann Arbor, MI) as we previously described [15]. Plasma obtained from 11 healthy subjects served as the normal control. The inclusion criteria for healthy subjects were: (1) adults aged from 20 to 65 years old; (2) not pregnant; and (3) do not have systemic disease.

### Statistical analysis

Data were expressed as mean ± standard deviation (SD) or as counts, as appropriate. The Mann–Whitney *U*-test was applied for assessing statistical significance of differences of between groups. Univariate and multivariate Cox regression analysis were performed to identify predictors for clinical outcomes. The results of the Cox regression analysis were reported as *p* value, hazard ratio (HR), and 95% confidence interval (CI). The cut-off values of MELD score, Child-Pugh score, M2BPGi, Fibrosis-4 (FIB-4) scores, and AST to platelet ratio index (APRI) scores in Cox regression analysis were determined by using median levels and the cut-off value of HVPG was according to the previous study [16]. Correlation between continuous variables was analyzed using Spearman’s correlation test and Spearman’s correlation coefficient was expressed as r_s_. Statistical significance was defined as *p* ≤ 0.05. All statistical analysis were performed using IBM SPSS Statistics for Windows, Version 24.0 (IBM Corp. Armonk, NY, USA).

## Results

### Patient characteristics

A total of 48 cirrhotic patients were retrospectively enrolled in this study, which included 36 males and 12 females with a mean age of 62.7 years. All the patients have portal hypertension (HVPG at or above 6mmHg) and 46 (95.8%) patients have clinically significant portal hypertension (HVPG at or above 10mmHg), with the mean HVPG of 17.5 mmHg. The demographic, laboratory and clinical characteristics are summarized in **Table 1**.

**Table 1:** Patient characteristics.

Variables	
**Age, years**	62.71 ± 12.54
**Male, *n* (%)**	36 (75.0)
**Etiology of cirrhosis, *n* (%)**	
Hepatitis B	23 (47.9)
Hepatitis C	13 (27.1)
Hepatitis B and C	4 (8.3)
Alcohol	2 (4.2)
Others^†^	6 (12.5)
**Anti-viral therapy, *n* (%)**	
Hepatitis B/Hepatitis C	6 (12.5)/0 (0)
**NSBB use after HVPG measurement, *n* (%)**	31 (64.6)
**Laboratory**	
Platelet (1000/uL)	8.21 ± 4.16
Sodium (mEq/L)	138.98 ± 3.22
Creatinine (mg/dL)	1.06 ± 0.27
Total bilirubin (mg/dL)	2.12 ± 2.20
Albumin (g/dL)	3.37 ± 0.59
INR	1.23 ± 0.20
M2BPGi (C.O.I)	7.19 ± 4.44
**Hemodynamics**	
HVPG (mmHg)	17.46 ± 4.38
HR (b.p.m.)	76.57 ± 12.44
CO (L/min)	6.61 ± 1.96
MAP (mmHg)	95.00 ± 10.80
SVR (dynes•s/cm^5^)	1247.95 ± 429.51
**ALBI scores**	-1.91 ± 0.60
**ALBI grade, *n* (%)**	
1/2/3	7 (14.6)/ 30 (62.5)/11 (22.9)
**Child-Pugh scores**	7.17 ± 2.05
**Child-Pugh classification, *n* (%)**	
A/B/C	23 (47.9)/17 (35.4)/8 (16.7)
**MELD scores**	11.28 ± 3.78
**FIB-4**	6.91 ± 3.95
**APRI**	1.31 ± 0.84
**Complication of cirrhosis at follow-up, *n* (%)**	
Ascites	17 (35.4)
Spontaneous bacterial peritonitis	14 (29.2)
Hepatorenal syndrome	9 (18.8)
Esophageal variceal bleeding	8 (16.7)
Hepatic encephalopathy	23 (47.9)
Hepatocellular carcinoma	15 (31.3)

The data are expressed as mean ± standard deviation or number (percent).

^†^Other etiologies of cirrhosis: three patients had cryptogenic cirrhosis, one patient had Wilson disease and one patient had primary biliary cholangitis.

NSBB, non-selective beta-blocker; INR, international normalized ratio; M2BPGi, Mac-2 binding protein glycosylation isomer; HVPG, hepatic venous pressure gradient; HR, heart rate; CO, cardiac output; MAP, mean arterial pressure; SVR, systemic vascular resistance; ALBI, Albumin-Bilirubin; MELD, Model for End-stage Liver Disease; FIB-4, Fibrosis-4; APRI, AST to platelet ratio index.

### Association between M2BPGi and HVPG in liver cirrhosis

**Fig 1:**
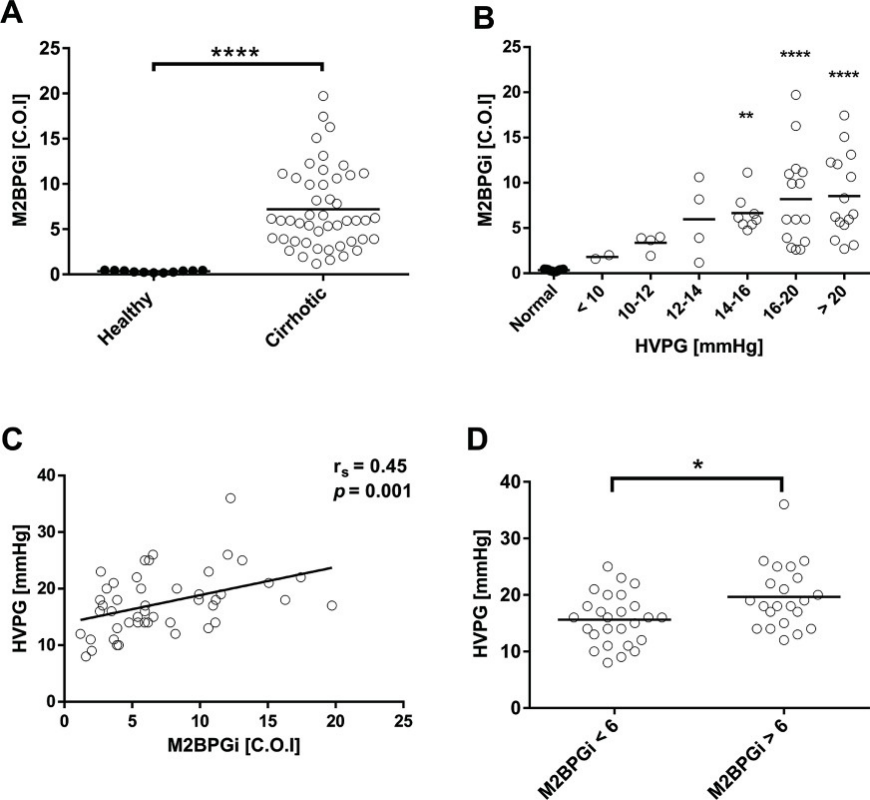
The association of M2BPGi with HVPG in cirrhotic patients with portal hypertension. (A) Comparison of plasma M2BPGi levels in healthy subjects versus cirrhotic patients; (B) Plasma M2BPGi levels in patients with different levels of HVPG; (C) Correlation between plasma M2BPGi levels and HVPG levels and (D) HVPG levels in patients with plasma M2BPGi ≥ 6 C.O.I. vs. < 6 C.O.I. *p < 0.05 vs. normal groups; **p < 0.01 vs. normal group; ****p < 0.0001 vs. normal group; M2BPGi, Mac‑2 binding protein glycosylation isomer; HVPG, hepatic venous pressure gradient.

Plasma M2BPGi levels were significantly higher in cirrhotic patients compared to healthy subjects (7.19 ± 4.44 vs. 0.34 ± 0.11, *p* < 0.001) **(Fig 1A)**. Interestingly, M2BPGi levels increased with the progression of portal hypertension **(Fig 1B)** in cirrhotic patients. Correlation between M2BPGi and HVPG was statistically significant (r_s_ = 0.45, *p* = 0.001) **(Fig 1C)**. However, correlation between M2BPGi and other hemodynamic parameters, including MAP, CO, and SVR, were not found. The median plasma level of M2BPGi was 6 C.O.I. in our study. Cirrhotic patients were then divided into group with M2BPGi ≥ 6 C.O.I. and group with M2BPGi < 6 C.O.I. The HVPG levels between the two groups were compared **(Fig 1D)**. Patients with plasma M2BPGi ≥ 6 C.O.I. had significantly higher HVPG levels than those with M2BPGi < 6 C.O.I. We also examined the association between HVPG and FIB-4 scores as well as APRI scores and found that HVPG levels did not correlated with both parameters (r_s_ = 0.09, *p* = 0.556 and r_s_ = 0.12, *p* = 0.404, respectively).

**Fig 2:**
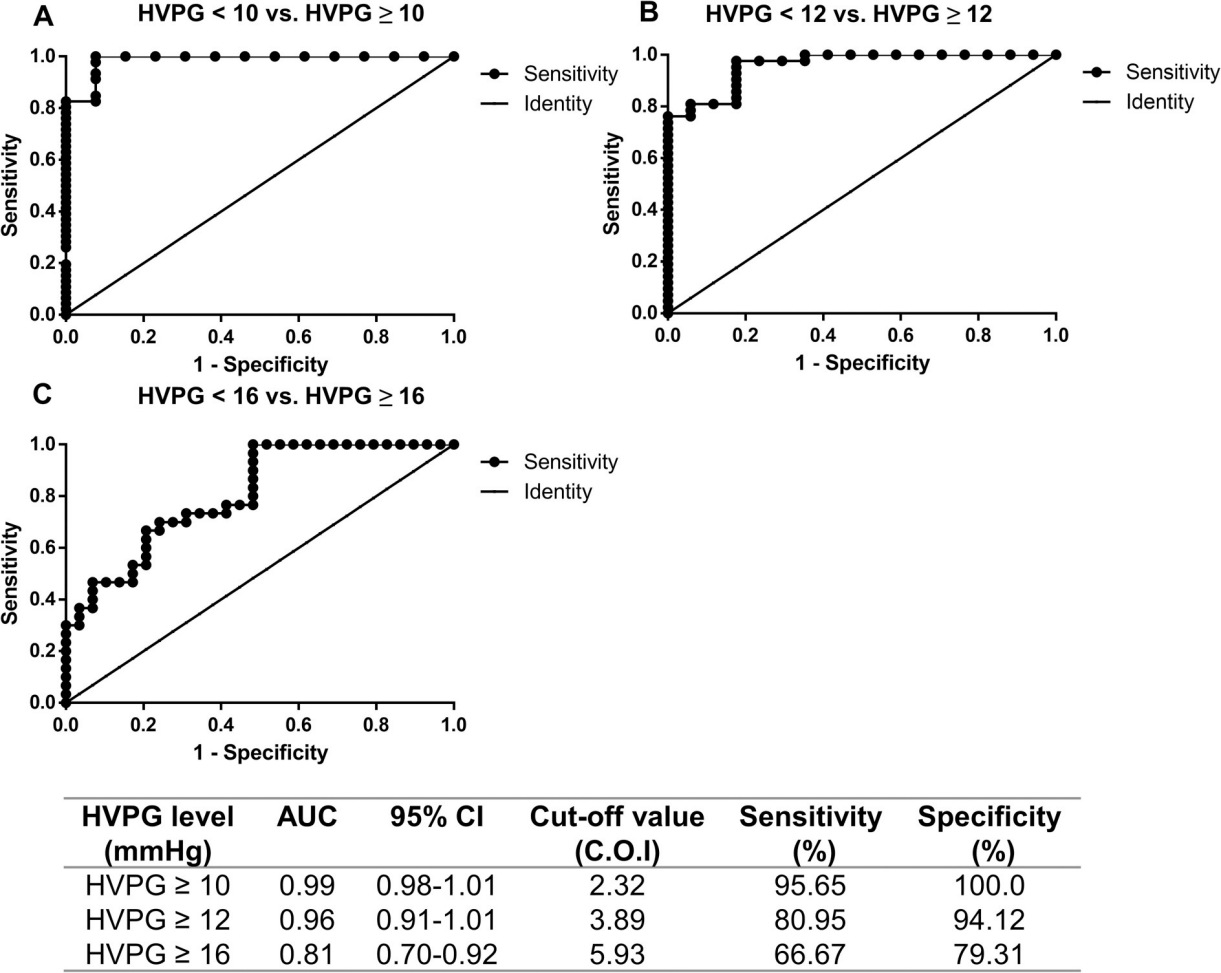
The areas under ROC curve of M2BPGi for distinguishing cirrhotic patients with (A) HVPG < 10 mmHg vs. HVPG ≥ 10 mmHg, (B) HVPG < 12 mmHg vs. HVPG ≥ 12 mmHg, and (C) HVPG < 16 mmHg vs. HVPG ≥ 16 mmHg. M2BPGi, Mac‑2 binding protein glycosylation isomer; HVPG, hepatic venous pressure gradient; ROC, receiver operating characteristic.

Diagnostic performance of M2BPGi for identifying patients with different levels of HVPG is shown in **Fig 2**. Areas under the curve for the diagnosis of HVPG ≥ 10mmHg, ≥ 12mmHg, and ≥ 16mmHg were 0.99 (95% CI = 0.98–1.01), 0.96 (95% CI = 0.91–1.01) and 0.81 (95% CI = 0.70–0.92), respectively. The most predictive cut-off values of M2BPGi to identify patients with HVPG ≥ 10mmHg, ≥ 12mmHg, and ≥ 16mmHg were 2.32, 3.89 and 5.93 C.O.I, respectively.

### Role of M2BPGi for predicting clinical outcomes in cirrhotic patients with portal hypertension

The median follow-up time was 20.1 (0.7–60.0) months. During the follow-up period, 32 patients died. Cox regression analysis was used to evaluate potential predictors associated with mortality within five years **(Table 2)**. On univariate analysis, higher Child-Pugh scores (≥ 7) and higher plasma levels of M2BPGi (≥ 6 C.O.I) were significant predictors for mortality **(Table 2 and S1 Fig)**. However, these parameters failed to predict mortality on multivariate analysis. The results were similar after excluding 14 patients with hepatocellular carcinoma **(S1 Table)**. Additionally, FIB-4 and APRI did not show a predictive role for mortality in these patients.

**Table 2:** Univariate and multivariate analysis for predictors of mortality.

Predictors		Univariate analysis			Multivariate analysis		
	*n*	HR	95%CI	*p*-value	HR	95%CI	*p*-value
Age (≥ 65/ < 65 years)	26/22	1.77	0.85–3.64	0.119			
Gender (male/female)	36/12	0.96	0.40–2.38	0.956			
HVPG (≥ 16/ < 16 mmHg)	30/18	1.96	0.91–4.26	0.088	1.48	0.65–3.36	0.352
MELD scores (≥ 11/ < 11)	22/26	1.85	0.92–3.74	0.085	1.11	0.47–3.64	0.817
Child-Pugh scores (≥ 7/ < 7)	25/23	2.28	1.11–4.68	0.025	1.40	0.51–3.83	0.515
M2BPGi (≥ 6/ < 6)	22/26	2.48	1.21–5.08	0.013	1.74	0.72–4.22	0.222
ALBI grade (3/1 and 2)	11/37	1.79	0.85–3.80	0.127			
FIB-4 (≥ 6/ < 6)	25/23	0.88	0.44–1.77	0.724			
APRI (≥ 1.3/ < 1.3)	21/27	0.89	0.44–1.81	0.754			
NSBB use	31/17	0.67	0.33–1.34	0.257			

HR, hazard ratio; CI, confidence interval; HVPG, hepatic venous pressure gradient; MELD, Model of End-Stage Liver Disease; M2BPGi, Mac-2 binding protein glycosylation isomer; ALBI, Albumin-Bilirubin; FIB-4, Fibrosis-4; APRI, AST to platelet ratio index; NSBB, non-selective beta-blocker.

**Fig 3:**
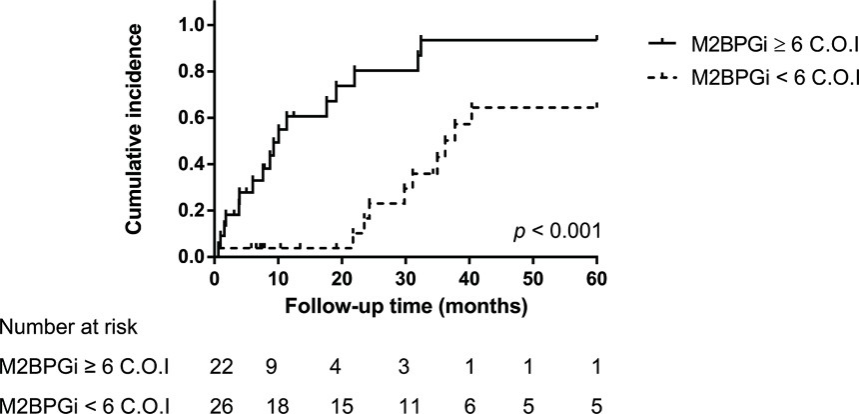
Cumulative incidence of developing bacterial infection during the follow-up period in patients with plasma Mac-2 binding protein glycosylation isomer levels above or below 6 C.O.I. The *p*-value corresponds to log–rank test. M2BPGi, Mac‑2 binding protein glycosylation isomer.

We further examined potential parameters to predict cirrhosis-related complications in patients with portal hypertension. Twenty-seven patients developed bacterial infection events, including bacteremia (n = 9), pneumonia (n = 2), urinary tract infection (n = 6), soft tissue infection (n = 3), and SBP (n = 11). Of the 11 patients with SBP, 7 patients suffered from both SBP and other infection events. On univariate analysis, Child-Pugh scores ≥ 7, albumin ≥ 3.5 g/dL and plasma M2BPGi levels ≥ 6 C.O.I were significant predictors for occurrence of bacterial infection within five years. However, on multivariate Cox regression analysis, plasma M2BPGi levels ≥ 6 C.O.I was the only significant predictor for bacterial infection events (HR = 4.51, *p* = 0.003) **(Table 3 and Fig 3)**. Nevertheless, none of these parameters showed a predictive value for occurrence of SBP and other cirrhosis-related complications **(S2–S6 Tables)**.

**Table 3:** Univariate and multivariate analysis for predictors of occurrence of bacterial infection.

Predictors		Univariate analysis			Multivariate analysis		
	*n*	HR	95%CI	*p*-value	HR	95%CI	*p*-value
Age (≥ 65/ < 65 years)	26/22	1.03	0.48–2.20	0.947			
Gender (male/female)	36/12	0.72	0.28–1.83	0.486			
HVPG (≥ 16/ < 16 mmHg)	30/18	1.69	0.75–3.80	0.202			
MELD scores (≥ 11/ < 11)	22/26	1.69	0.78–3.63	0.181			
Child-Pugh scores (≥ 7/ < 7)	25/23	2.68	1.22–5.89	0.014	1.33	0.44–3.97	0.615
M2BPGi (≥ 6/ < 6)	22/26	5.57	2.32–13.35	<0.001	4.51	1.68–12.11	0.003
ALBI grade (3/1 and 2)	11/37	1.98	0.82–4.74	0.127			
FIB-4 (≥ 6/ < 6)	25/23	0.71	0.34–1.48	0.360			
APRI (≥ 1.3/ < 1.3)	21/27	0.70	0.34–1.46	0.347			
NSBB use	31/17	1.09	0.50–2.40	0.823			
Albumin (≥ 3.5/ < 3.5g/dL)	21/27	0.38	0.17–0.88	0.024	0.84	0.26–2.70	0.767
Presence of ascites at diagnosis	16/32	1.34	0.56–3.21	0.509			

HR, hazard ratio; CI, confidence interval; HVPG, hepatic venous pressure gradient; MELD, Model of End-Stage Liver Disease; M2BPGi, Mac-2 binding protein glycosylation isomer; ALBI, Albumin-Bilirubin; FIB-4, Fibrosis-4; APRI, AST to platelet ratio index; NSBB, non-selective beta-blocker.

We further examined the association of M2BPGi and pro-inflammatory cytokines including IL-1α, IL-1β, IL-1Ra, endotoxin and nitric oxide in these patients. We found that M2BPGi was positively correlated with IL-1α (r_s_ = 0.46, *p* = 0.002) and IL-1Ra (r_s_ = 0.32, *p* = 0.042). There was no significant correlation between M2BPGi and other pro-inflammatory cytokines, including IL-1β (r_s_ = 0.30, *p* = 0.068), endotoxin (r_s_ = -0.11, *p* = 0.514) and nitric oxide (r_s_ = 0.27, *p* = 0.103).

## Discussion

In the present study, we demonstrated that plasma levels of M2BPGi correlated with HVPG levels in cirrhotic patients. Furthermore, bacterial infection events occurred at higher rates in those with higher plasma M2BPGi concentrations, suggesting that M2BPGi could be a new prognostic marker for bacterial infection for cirrhotic patients with portal hypertension. The results in our study provide novel information that may help clinical physicians to identify patients who are benefit from aggressive surveillance of cirrhosis-related complications.

It is known that HVPG levels correlate with the degree of fibrosis on liver histology [17–19] and has been used to predict clinical outcomes among cirrhotic patients. However, the utility of HVPG measurement is limited in clinical use owing to the invasiveness. Therefore, numerous studies aim to find a non-invasive tool which is highly accurate, repeatable, and convenient in clinical use. Recently, M2BPGi, an altered form of Mac-2 binding protein (M2BP) due to changes in the N-glycosylation during the progression of hepatic fibrosis, has been reported as a novel biomarker for liver fibrosis [7]. Although studies have confirmed the diagnostic accuracy of M2BPGi for liver fibrosis with various etiologies, especially for advanced fibrosis [8,20], no studies have investigated the association of M2BPGi and portal pressure in cirrhotic patients. In the present study, we found that there is a positive correlation between M2BPGi and HVPG in cirrhotic patients. Importantly, we identified cut-off values of M2BPGi to stratify patients with different degree of portal hypertension: 2.33 C.O.I for patients with clinical significantly portal hypertension (HVPG ≥ 10mmHg), 3.89 C.O.I for patients with severe portal hypertension (HVPG ≥ 12mmHg) and 5.93 C.O.I for patients with increased risk of mortality (HVPG ≥ 16mmHg). The findings in our study suggest that M2BPGi might play a role in recognizing cirrhotic patients with different severity of portal hypertension.

Additionally, previous studies have demonstrated that plasma M2BPGi levels were higher in patients with decompensated cirrhosis and higher plasma M2BPGi level was an independent risk factor for liver-related mortality or all-cause mortality in cirrhotic patients [21–24]. Nevertheless, in our studies, we found that M2BPGi did not show a predictive value for all-cause mortality and occurrence of cirrhosis-related complications, including SBP, hepatic encephalopathy, variceal bleeding, hepatorenal syndrome during the follow-up period. One of the reasons for the discrepant results may be attributed to the different enrolled populations between studies. In our study, 95.8% of patientshave clinically significant portal hypertension with the mean HVPG of 17.5 mmHg, which indicated that most patients enrolled in our study had more advanced fibrosis and were prone to have poor outcomes and higher mortality, leading to the different results as compared with the other studies. Furthermore, the different cut-off values of M2BPGi between studies and a small patient population in the present study might also contribute to different results.

An interesting finding in the present study was that M2BPGi was a potential predictor for the occurrence of bacterial infection. Plasma M2BPGi levels were also associated with plasma IL-1α and IL-1Ra levels. Although there was no statistic correlation between M2BPGi and IL-1β, there was a trend between the parameters. Bacterial infection is a troublesome consequence of cirrhosis, because in the course of advanced cirrhosis, patients encounter cirrhosis-associated immune dysfunction, with impaired immune system and dysregulated immune cell activation [25]. Bacterial-derived toxins in cirrhotic patients may trigger the production of proinflammatory cytokines from macrophage or monocytes. In vitro, human M2BP induces production of IL-1and other cytokines by blood monocytes [26]. IL-1, which includes IL-1α and IL-1β, is a central mediator of innate immunity and inflammation. The proinflammatory activities of IL-1 are controlled by several endogenous inhibitors and one of which is IL-1 receptor antagonist (IL-1Ra) [27]. In our previous study, we demonstrated that IL-1α, IL-1β and IL-1Ra were increased in cirrhotic patients and increased IL-1Ra levels predicted bacterial infection events [13]. As elevated IL-1 and IL-1Ra might reflect the immunodeficient status and dysregulated immune cell activation in cirrhotic patients, higher plasma concentration of M2BPGi might also reflect an immunosuppressive condition and these patients are susceptible to bacterial infection. Nevertheless, M2BPGi failed to predict the development of SBP on multivariate analysis (S4 Table), which was an important type of bacterial infection for cirrhotic patients. The possible reasons might be attributed to the small sample size and limited events in our study. High mortality rate in our studies might also contribute a role because some patients might die due to other complications before occurrence of SBP. Further prospective studies with larger sample size and longer follow-up time are needed to elucidate the diagnostic accuracy of M2BPGi in predicting HVPG levels and bacterial infection, especially SBP, in cirrhotic patients.

This study has some limitations. First, this is a retrospective study using data from a single medical center with small sample size, and caution must be taken in interpreting data. Second, patients with hepatic encephalopathy, active infection and active bleeding were excluded at enrollment and information about these patient populations is lacking. Furthermore, most of the patients had clinically significant portal hypertension, which indicates advanced cirrhosis in these patients. Therefore, the findings in our study might only be applied to cirrhotic patients with advanced portal hypertension.

In conclusion, plasma levels of M2BPGi correlated with HVPG levels and might predict severity of portal hypertension in cirrhotic patients. Additionally, higher plasma levels of M2BPGi could help to predict the occurrence of bacterial infection. The results in this study suggest that M2BPGi is a potential biomarker that could provide information to clinical physicians about the presence and severity of portal hypertension in cirrhotic patients and help them to identify patients who are at risk of developing bacterial infection.

## Supporting information

S1 Fig Cumulative survival rate of mortality during the follow-up period in patients with plasma Mac-2 binding protein glycosylation isomer levels above or below 6 C.O.I. The *p*-value corresponds to log–rank test. M2BPGi, Mac‑2 binding protein glycosylation isomer.
(DOCX)

S1 Table Univariate and multivariate analysis for predictors of mortality after excluding patients with hepatocellular carcinoma. (DOCX)

S2 Table Univariate and multivariate analysis for predictors of occurrence of hepatorenal syndrome. (DOCX)

S3 Table Univariate and multivariate analysis for predictors of developing ascites. (DOCX)

S4 Table Univariate and multivariate analysis for predictors of spontaneous bacterial peritonitis. (DOCX)

S5 Table Univariate and multivariate analysis for predictors of hepatic encephalopathy. (DOCX)

S6 Table Univariate and multivariate analysis for predictors of esophageal variceal bleeding. (DOCX)
