# Supplementary material for: Mac-2 binding protein glycosylation isomer is a potential biomarker to predict portal hypertension and bacterial infection in cirrhotic patients
Source: PLoS One. 2021 Oct 14;16(10):e0258589. doi: 10.1371/journal.pone.0258589 (PMC8516253; doi:10.1371/journal.pone.0258589)
Supplement: S1 Fig — The p-value corresponds to log–rank test. M2BPGi, Mac‑2 binding protein glycosylation isomer. (DOCX) [file pone.0258589.s001.docx]

**S1 Figure.**


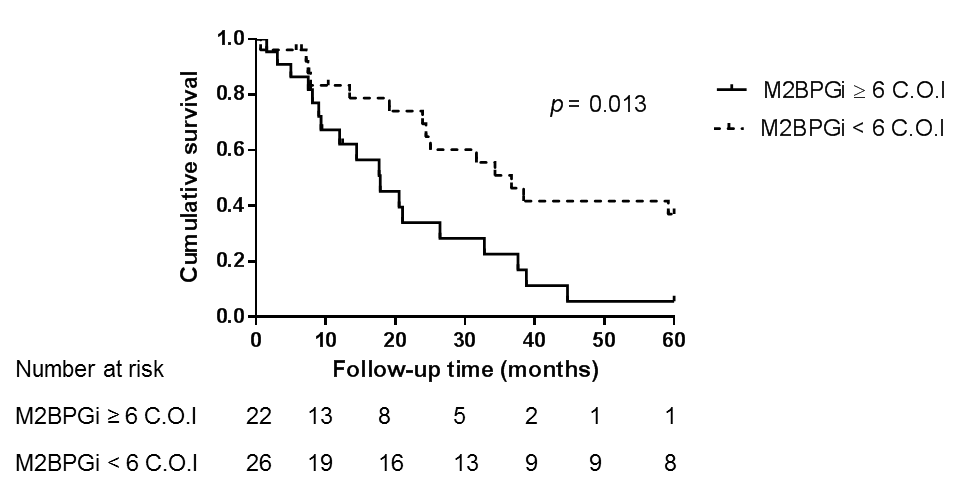


**S1 Figure.** Cumulative survival rate during the follow-up period in patients with plasma Mac-2 binding protein glycosylation isomer levels above or below 6 C.O.I. The *p*-value corresponds to log–rank test. M2BPGi, Mac‑2 binding protein glycosylation isomer.
