# Supplementary material for: Mac-2 binding protein glycosylation isomer is a potential biomarker to predict portal hypertension and bacterial infection in cirrhotic patients
Source: PLoS One. 2021 Oct 14;16(10):e0258589. doi: 10.1371/journal.pone.0258589 (PMC8516253; doi:10.1371/journal.pone.0258589)
Supplement: S1 Table — (DOCX) [file pone.0258589.s002.docx]

| Predictors |  | Univariate analysis | | | Multivariate analysis | | |
| --- | --- | --- | --- | --- | --- | --- | --- |
|  | ***n*** | **HR** | **95%CI** | ***p*-value** | **HR** | **95%CI** | ***p*-value** |
| Age ( ≥ 65/ < 65 years) | 16/18 | 1.95 | 0.87-4.35 | 0.103 |  |  |  |
| Gender (male/female) | 24/10 | 0.97 | 0.38-2.44 | 0.940 |  |  |  |
| HVPG ( ≥ 16/ < 16mmHg) | 24/10 | 1.16 | 0.46-2.91 | 0.755 |  |  |  |
| MELD scores ( ≥ 11/ < 11) | 17/17 | 1.57 | 0.70-3.51 | 0.275 |  |  |  |
| Child-Pugh scores ( ≥ 7 / < 7) | 20/14 | 2.34 | 0.9-5.47 | 0.050 | 1.96 | 0.75-5.11 | 0.169 |
| M2BPGi ( ≥ 6/ < 6) | 1816 | 1.52 | 0.68-3.39 | 0.302 |  |  |  |
| ALBI grade (3/1 and 2) | 8/26 | 2.24 | 0.93-5.30 | 0.068 | 1.55 | 0.59-4.09 | 0.374 |
| FIB-4 ( ≥ 6/ < 6) | 17/17 | 0.67 | 0.30-1.48 | 0.317 |  |  |  |
| APRI ( ≥ 1.3/ < 1.3) | 13/21 | 0.83 | 0.36-1.89 | 0.655 |  |  |  |

**S1 Table. Univariate and multivariate analysis for predictors of mortality after excluding patients with hepatocellular carcinoma**

HR, hazard ratio; CI, conﬁdence interval; HVPG, hepatic venous pressure gradient; MELD, Model of End-Stage Liver Disease; M2BPGi, Mac-2 binding protein glycosylation isomer; ALBI, Albumin-Bilirubin; FIB-4, Fibrosis-4; APRI, AST to platelet ratio index
