# Supplementary material for: Mac-2 binding protein glycosylation isomer is a potential biomarker to predict portal hypertension and bacterial infection in cirrhotic patients
Source: PLoS One. 2021 Oct 14;16(10):e0258589. doi: 10.1371/journal.pone.0258589 (PMC8516253; doi:10.1371/journal.pone.0258589)
Supplement: S2 Table — (DOCX) [file pone.0258589.s003.docx]

**S2 Table. Univariate and multivariate analysis for predictors of occurrence of hepatorenal syndrome**

| Predictors |  | Univariate analysis | | | Multivariate analysis | | |
| --- | --- | --- | --- | --- | --- | --- | --- |
|  | ***n*** | **HR** | **95%CI** | ***p*-value** | **HR** | **95%CI** | ***p*-value** |
| Age ( ≥ 65/ < 65 years) | 26/22 | 2.12 | 0.39-11.67 | 0.387 |  |  |  |
| Gender (male/female) | 36/12 | 1.04 | 0.12-9.04 | 0.970 |  |  |  |
| HVPG ( ≥ 16/ < 16mmHg) | 30/18 | 2.92 | 0.46-33.72 | 0.213 |  |  |  |
| MELD scores ( ≥ 11/ < 11) | 22/26 | 7.40 | 0.86-63.91 | 0.069 | 3.23 | 0.31-33.73 | 0.328 |
| Child-Pugh scores ( ≥ 7/ < 7) | 25/23 | 2.86 | 0.52-15.70 | 0.226 |  |  |  |
| M2BPGi ( ≥ 6/ < 6) | 22/26 | 10.47 | 1.20-91.43 | 0.034 | 6.09 | 0.58-64.22 | 0.133 |
| ALBI grade (3/1 and 2) | 11/37 | 0.80 | 0.093-6.87 | 0.838 |  |  |  |
| FIB-4 ( ≥ 6/ < 6) | 25/23 | 56.99 | 0.08-41837.96 | 0.230 |  |  |  |
| APRI ( ≥ 1.3/ < 1.3) | 21/27 | 2.55 | 0.46-13.97 | 0.282 |  |  |  |

HR, hazard ratio; CI, conﬁdence interval; HVPG, hepatic venous pressure gradient; MELD, Model of End-Stage Liver Disease; M2BPGi, Mac-2 binding protein glycosylation isomer; ALBI, Albumin-Bilirubin; FIB-4, Fibrosis-4; APRI, AST to platelet ratio index
