# Supplementary material for: Mac-2 binding protein glycosylation isomer is a potential biomarker to predict portal hypertension and bacterial infection in cirrhotic patients
Source: PLoS One. 2021 Oct 14;16(10):e0258589. doi: 10.1371/journal.pone.0258589 (PMC8516253; doi:10.1371/journal.pone.0258589)
Supplement: S3 Table — (DOCX) [file pone.0258589.s004.docx]

**S3 Table. Univariate and multivariate analysis for predictors of developing ascites**

| Predictors |  | Univariate analysis | | |
| --- | --- | --- | --- | --- |
|  | ***n*** | **HR** | **95%CI** | ***p*-value** |
| Age ( ≥ 65/ < 65 years) | 26/22 | 2.87 | 0.93-8.55 | 0.058 |
| Gender (male/female) | 36/12 | 0.72 | 0.20-2.66 | 0.625 |
| HVPG ( ≥ 16/ < 16mmHg) | 30/18 | 1.65 | 0.56-4.88 | 0.367 |
| MELD scores ( ≥ 11/ < 11) | 22/26 | 0.73 | 0.33-2.63 | 0.897 |
| Child-Pugh scores ( ≥ 7/ < 7) | 25/23 | 0.64 | 0.22-1.87 | 0.413 |
| M2BPGi ( ≥ 6/ < 6) | 22/26 | 1.47 | 0.52-4.12 | 0.467 |
| ALBI grade (3/1 and 2) | 11/37 | 0.23 | 0.03-1.74 | 0.154 |
| FIB-4 ( ≥ 6/ < 6) | 25/23 | 0.70 | 0.25-1.92 | 0.483 |
| APRI ( ≥ 1.3/ < 1.3) | 21/27 | 0.74 | 0.26-2.07 | 0.560 |

HR, hazard ratio; CI, conﬁdence interval; HVPG, hepatic venous pressure gradient; MELD, Model of End-Stage Liver Disease; M2BPGi, Mac-2 binding protein glycosylation isomer; ALBI, Albumin-Bilirubin; FIB-4, Fibrosis-4; APRI, AST to platelet ratio index
