# Supplementary material for: Mac-2 binding protein glycosylation isomer is a potential biomarker to predict portal hypertension and bacterial infection in cirrhotic patients
Source: PLoS One. 2021 Oct 14;16(10):e0258589. doi: 10.1371/journal.pone.0258589 (PMC8516253; doi:10.1371/journal.pone.0258589)
Supplement: S4 Table — (DOCX) [file pone.0258589.s005.docx]

**S4 Table. Univariate and multivariate analysis for predictors of spontaneous bacterial peritonitis**

| Predictors |  | Univariate analysis | | | Multivariate analysis | | |
| --- | --- | --- | --- | --- | --- | --- | --- |
|  | ***n*** | **HR** | **95%CI** | ***p*-value** | **HR** | **95%CI** | ***p*-value** |
| Age ( ≥ 65/ < 65 years) | 26/22 | 0.58 | 0.17-2.00 | 0.386 |  |  |  |
| Gender (male/female) | 36/12 | 0.68 | 0.18-2.58 | 0.567 |  |  |  |
| HVPG ( ≥ 16/ < 16mmHg) | 30/18 | 7.70 | 0.98-60.33 | 0.052 | 4.86 | 0.58-40.70 | 0.145 |
| MELD scores ( ≥ 11/ < 11) | 22/26 | 1.84 | 0.56-6.09 | 0.316 |  |  |  |
| Child-Pugh scores ( ≥ 7/ < 7) | 25/23 | 3.75 | 0.99-14.20 | 0.052 | 1.37 | 0.27-6.90 | 0.700 |
| M2BPGi ( ≥ 6/ < 6) | 22/26 | 5.57 | 1.44-21.58 | 0.013 | 3.23 | 0.64-16.27 | 0.156 |
| ALBI grade (3/1 and 2) | 11/37 | 2.39 | 0.70-8.18 | 2.385 |  |  |  |
| FIB-4 ( ≥ 6/ < 6) | 25/23 | 1.01 | 0.31-3.33 | 0.982 |  |  |  |
| APRI ( ≥ 1.3/ < 1.3) | 21/27 | 1.09 | 0.33-3.59 | 0.884 |  |  |  |

HR, hazard ratio; CI, conﬁdence interval; HVPG, hepatic venous pressure gradient; MELD, Model of End-Stage Liver Disease; M2BPGi, Mac-2 binding protein glycosylation isomer; ALBI, Albumin-Bilirubin; FIB-4, Fibrosis-4; APRI, AST to platelet ratio index
