# Supplementary material for: Mac-2 binding protein glycosylation isomer is a potential biomarker to predict portal hypertension and bacterial infection in cirrhotic patients
Source: PLoS One. 2021 Oct 14;16(10):e0258589. doi: 10.1371/journal.pone.0258589 (PMC8516253; doi:10.1371/journal.pone.0258589)
Supplement: S5 Table — (DOCX) [file pone.0258589.s006.docx]

**S5 Table. Univariate and multivariate analysis for predictors of hepatic encephalopathy**

| Predictors |  | Univariate analysis | | | Multivariate analysis | | |
| --- | --- | --- | --- | --- | --- | --- | --- |
|  | ***n*** | **HR** | **95%CI** | ***p*-value** | **HR** | **95%CI** | ***p*-value** |
| Age ( ≥ 65/ < 65 years) | 26/22 | 1.36 | 0.57-3.24 | 0.485 |  |  |  |
| Gender (male/female) | 36/12 | 0.77 | 0.28-2.13 | 0.611 |  |  |  |
| HVPG ( ≥ 16/ < 16mmHg) | 30/18 | 2.21 | 0.85-5.75 | 0.104 |  |  |  |
| MELD scores ( ≥ 11 / < 11) | 22/26 | 2.38 | 0.99-5.74 | 0.053 | 1.51 | 0.53-4.36 | 0.443 |
| Child-Pugh scores ( ≥ 7/ < 7) | 25/23 | 2.53 | 1.04-6.15 | 0.041 | 1.54 | 0.49-4.89 | 0.463 |
| M2BPGi ( ≥ 6 / < 6) | 22/26 | 2.47 | 1.02-5.95 | 0.044 | 1.60 | 0.55-4.64 | 0.390 |
| ALBI grade (3/1 and 2) | 11/37 | 1.04 | 0.35-3.10 | 0.949 |  |  |  |
| FIB-4 ( ≥ 6/ < 6) | 25/23 | 1.38 | 0.58-3.28 | 0.469 |  |  |  |
| APRI ( ≥ 1.3/ < 1.3) | 21/27 | 1.07 | 0.45-2.55 | 0.882 |  |  |  |

HR, hazard ratio; CI, conﬁdence interval; HVPG, hepatic venous pressure gradient; MELD, Model of End-Stage Liver Disease; M2BPGi, Mac-2 binding protein glycosylation isomer; ALBI, Albumin-Bilirubin; FIB-4, Fibrosis-4; APRI, AST to platelet ratio index
