# Supplementary material for: Mac-2 binding protein glycosylation isomer is a potential biomarker to predict portal hypertension and bacterial infection in cirrhotic patients
Source: PLoS One. 2021 Oct 14;16(10):e0258589. doi: 10.1371/journal.pone.0258589 (PMC8516253; doi:10.1371/journal.pone.0258589)
Supplement: S6 Table — (DOCX) [file pone.0258589.s007.docx]

**S6 Table. Univariate and multivariate analysis for predictors of esophageal variceal bleeding**

| Predictors |  | Univariate analysis | | |
| --- | --- | --- | --- | --- |
|  | ***n*** | **HR** | **95%CI** | ***p*-value** |
| Age ( ≥ 65/ < 65 years) | 26/22 | 0.49 | 0.09-2.71 | 0.416 |
| Gender (male/female) | 36/12 | 0.488 | 0.09-2.68 | 0.409 |
| HVPG ( ≥ 16/ < 16mmHg) | 30/18 | 1.39 | 0.25-7.58 | 0.707 |
| MELD scores ( ≥ 11 / < 11) | 22/26 | 1.15 | 0.23-5.71 | 0.864 |
| Child-Pugh scores ( ≥7/ < 7) | 25/23 | 2.74 | 0.50-15.05 | 0.246 |
| M2BPGi ( ≥ 6 / < 6) | 22/26 | 1.75 | 0.35-8.9 | 0.500 |
| ALBI grade (3/1 and 2) | 11/37 | 4.27 | 0.86-21.24 | 0.077 |
| FIB-4 ( ≥ 6/ < 6) | 25/23 | 0.81 | 0.16-4.04 | 0.795 |
| APRI ( ≥ 1.3/ < 1.3) | 21/27 | 0.65 | 0.12-3.59 | 0.624 |

HR, hazard ratio; CI, conﬁdence interval; HVPG, hepatic venous pressure gradient; MELD, Model of End-Stage Liver Disease; M2BPGi, Mac-2 binding protein glycosylation isomer; ALBI, Albumin-Bilirubin; FIB-4, Fibrosis-4; APRI, AST to platelet ratio index
